# Secretory IgA in Mucosa of Pharynx and Larynx Plays an Important Role against Influenza A Virus Infection in Kidney Yang Deficiency Syndrome Model

**DOI:** 10.1155/2020/9316763

**Published:** 2020-03-29

**Authors:** Shaozhe Zhao, Lei Yuan, Yi Li, Longchan Liu, Zixin Luo, Qingtao Lv, Rong Rong, Yong Yang

**Affiliations:** ^1^Shandong University of Traditional Chinese Medicine, Jinan, Shandong 250355, China; ^2^Shandong Provincial Collaborative Innovation Center for Antiviral Traditional Chinese Medicine, Jinan, Shandong 250355, China

## Abstract

**Objective:**

Influenza virus poses a major threat to human health and has serious morbidity and mortality which commonly occurs in high-risk populations. Pharynx and larynx of the upper respiratory tract mucosa is the first defense line against influenza virus infection. However, the ability of the pharynx and larynx organ to eliminate the influenza pathogen is still not clear under different host conditions.

**Methods:**

In this study, a mouse model of kidney yang deficiency syndrome (KYDS) was used to mimic high-risk peoples. Two different methods of influenza A (H1N1) virus infection by nasal dropping or tracheal intubation were applied to these mice, which were divided into four groups: normal intubation (NI) group, normal nasal dropping (ND) group, model intubation (MI) group, and model nasal dropping (MD) group. The normal control (NC) group was used as a negative control. Body weight, rectal temperature, and survival rate were observed every day. Histopathologic changes, visceral index, gene expressions of H1N1, cytokine expressions, secretory IgA (SIgA) antibodies of tracheal lavage fluids in the upper respiratory tract, and bronchoalveolar lavage fluids were analyzed by ELISA.

**Results:**

The MD group had an earlier serious morbidity and mortality than the others. MI and NI groups became severe only in the 6^th^ to 7^th^ day after infection. The index of the lung increased significantly in NI, MI, and MD groups. Conversely, indices of the thymus and spleen increased significantly in NC and ND groups. H&E staining showed severe tissue lesions in MD, MI, and NI groups. H1N1 gene expressions were higher in the MD group compared with the MI group on the 3^rd^ day; however, the MD group decreased significantly on the 7^th^ day. IL-6 levels increased remarkably, and SIgA expressions decreased significantly in the MD group compared with the NC group.

**Conclusions:**

SIgA secretions are influenced directly by different conditions of the host in the pharynx and larynx in the upper respiratory tract mucosa. In the KYDS virus disease mode, SIgA expressions could be inhibited severely, which leads to serious morbidity and mortality after influenza A virus infection. The SIgA expressions of the pharynx and larynx would be an important target in high-risk populations against the influenza A virus for vaccine or antiviral drugs research.

## 1. Introduction

Influenza is a highly infectious disease that causes a worldwide public health problem in millions of peoples per year. The most common influenza A (H1N1) virus can cause seasonal infections with significant morbidity and mortality in elderly and high-risk adults all around the world [1–5]. It remains unclear why these high-risk populations have serious difficulties in the resolution of influenza virus infection and what factors affect the infectious outcome.

Kidney yang deficiency syndrome (KYDS) is one of the classical syndrome patterns in traditional Chinese medicine (TCM), and it reflects a constitutional tendency of elder peoples with weakness in the knees and lumbar regions, fatigue, difficulty in urination, enuresis, female sterility, reduction of ear functions, and tooth impairment [6–8]. It is the most popular syndrome that occurs in more than sixty-year-olds [6, 9–11]. The influenza virus could cause up to 90% mortality in this age group [12–15], and these susceptible individuals belong to high-risk peoples with influenza infection, which is mostly similar to the KYDS according to the principles of TCM [10, 16]. When an epidemiological investigation was conducted in 2137 healthy elderly above 60-year-old residents, the results showed that the incidence rate of kidney deficiency was 78.80% [16], and the kidney deficiency syndrome prevalence in participants showed an increasing trend with increasing age and deteriorating health status [17]. Another epidemiological study on 2,067 adults aged >60 years revealed that 45.33% suffered from KYDS, showing that KYDS is the predominant TCM syndrome in high-risk populations [18] and these elderly groups are at increased risk for serious flu complications. Therefore, a key challenge is how to reduce the high mortality in susceptible population infected with the influenza virus.

The mucous layer in the upper respiratory tract (pharynx and larynx) is the first innate barrier of defense against influenza virus infection. The virus replication is restricted by the first innate immune line [19, 20]. The defensive functional differences in the mucosal immunity of the pharynx and larynx between normal population and high-risk population are still unknown. As the influenza viral infections mostly have upper respiratory symptoms with sore throat or discomfort, we speculated that the mucosal immunity of the pharynx and larynx may play an important role against influenza virus infection in the initial stage. In order to test this hypothesis, we designed a special experiment by separate infection normal control mice and KYDS mice (by injecting estradiol benzoate intraperitoneally [21, 22]) combined with influenza A (H1N1) virus through nose dropping and tracheal injection, respectively. This experiment would be conductive to reveal the primary mechanism to combat viral infections in the mucosal immunity of the pharynx and larynx in the KYDS mice model.

## 2. Materials and Methods

### 2.1. Experimental Design

This study was approved by the Ethic Committee of Shandong University of Traditional Chinese Medicine, and all animals received humane care in compliance with the Chinese Animal Protection Act and the National Research Council Criteria. SPF Male BALB/c mice (16–18 g) were purchased from the Jinan Pengyue Experimental Animal Breeding Company Limited. After three days acclimation, all mice were randomly assigned to two groups: KYDS mice (model group) were established by intraperitoneal injection of estradiol benzoate (8 mg/kg, Ningbo Second Hormone Factory, China) for 7 d as previously described [21, 23] and normal control mice (normal group) were intraperitoneally injected with normal saline. The physical signs in mice were observed, and spontaneous activity, swimming time, rectal temperature, and body weight were monitored. All the animals were housed at 21–23°C in a 12 h light/12 h dark cycle, and environmental humidity was controlled between 60 and 70%.

After the KYDS mice model was finished, these model animals were randomly divided into two groups: model intubation (MI) group and model nasal dropping (MD) group. Normal group animals were divided into three groups: normal control (NC) group, normal intubation (NI) group, and normal nasal dropping (ND) group.

### 2.2. Virus

Mouse-adapted influenza A virus (A/FM/1/47, H1N1) was kindly donated by the Institute of Basic Medicine, Shandong Academy of Medical Sciences. The virus was amplified in the allantoic cavity of embryonated eggs at 36°C for 48 h with a hemagglutination titer of 1 : 320 and then stored at −80°C. All tests were performed in class II biosafety cabinets [21].

### 2.3. Visualized Method for Tracheal Intubation

We used mouse-sized speculum, a plastic incisor loop, and a rodent work stand (Hallowell Engineering and Manufacturing Corporation, USA) for the visualization procedure of tracheal intubation. The mouse-sized speculum is attached with an otoscope and has a side cut-away portion that is conducive to the ET tube and a mouse ET tube introducer passing through the trachea [24].

### 2.4. Tracheal Infection with Influenza A Virus (H1N1)

The intubation method was adapted from the operation guidelines by Hallowell EMC and previous literature [25–27]. Mice were anesthetized intraperitoneally (i.p.) with 500 mg·kg^−1^ tribromoethanol solution in the biosafety cabinet. Mice were placed on the work stand, and a clear view of the trachea was obtained with the otoscope and attached mouse speculum. The NI group and the MI group mice were injected with 5 *μ*L of viral suspension containing a hemagglutination titer of 1 : 320 of the influenza A/FM/1/47 (H1N1) virus per mouse through the ET tube (1.22 mm OD; 23.5 mm length). We have tested that the ET tube length position was just located in the upper respiratory tract below the oropharyngeal structure. The NC group mice were injected with the same volume normal saline.

### 2.5. Nasal Dropping Infection with Influenza A Virus (H1N1)

As a common nasal dropping infection method, the ND group and the MD group were anaesthetized intraperitoneally (i.p.) with 500 mg·kg^−1^ tribromoethanol solution and intranasally inoculated with 5 *μ*L and the same titer of the influenza A/FM/1/47 (H1N1) virus, and then they ate food and drank water freely.

### 2.6. Serum and Tissue Preparation

On the 14^th^ day, the whole blood was collected from the mice orbit and serum was later separated by centrifugalization, and then mice were sacrificed by cervical dislocation. The thymus, lung, and spleen were isolated and weighed for the organ index calculation. After harvesting, little amount of the right part of the lung samples was immediately submerged in RNAstore Reagent (Tiangen, China) at a dilution ratio of 1 : 10 (w/v) and then stored at 4°C. The left part of the lung tissue was taken, fixed in 10% formalin solution as histological specimens, and observed under the microscope to distinguish the pathological changes.

### 2.7. Cytometric Bead Array for Cytokine Measurements

Cytokine serum levels were determined using commercially available kits, including the mouse inflammation kit cytometric bead array (CBA; BD Biosciences Pharmingen, USA), to quantify IL-6, MCP-1, IL-12p70, and TNF-alpha. The CBA immunoassay uses 7.5 *μ*m polystyrene microbeads assembled in distinct fluorescent sets, unique on their type-4 fluorescence intensity (FL-4). Each microbead is coupled to the monoclonal antibody (MAb) against a given cytokine. Following incubation with the test sample, the bead-captured cytokines were detected by the direct immunoassay using a “detection cocktail” of distinct MAbs labeled with type-2 fluorescence, phycoerythrin-PE (FL-2). Data acquisition and analysis was performed in a dual-laser C6™ flow cytometer (BD Biosciences Pharmingen, San Jose, CA, USA), using the BD Bioscience CBA software. The fluorescently labeled particles in the BD CBA immunoassay are designed to be excited by the 488 nm and 532 nm lasers on the BD flow cytometer [28].

### 2.8. Total RNA Extraction

Total RNA was isolated from 10 to 20 mg tissues using RNAprep Pure Tissue Kit (Tiangen, China) following the manufacturer's instructions. Yield and purity of RNA were determined by the Quawell 5000 spectrophotometer (Quawell Technology, USA). RNA samples with an absorbance ratio OD 260/280 between 1.8 and 2.0 were used for further analysis. RNA integrity was assessed using agarose gel electrophoresis.

### 2.9. Reverse Transcription cDNA Synthesis

The first-strand cDNA was synthesized from 2 *μ*g of total RNA with random hexamer oligonucleotide primers using a 20 *μ*l reverse transcription system (FastQuant RT kit with gDNase, Tiangen, China) by incubation at 42°C for 3 min to protect the total RNA from genomic DNA interference. FastQuant RT Enzyme was used for reverse transcription at 42°C for 15 min. Then, cDNA was stored at −20°C for future use. For qPCR analysis, each cDNA sample was diluted 10 times with nuclease-free water.

### 2.10. Real-Time PCR

Real-time PCRs were conducted in Bio-Rad CFX Connect Real-Time System. For each reaction, the 20 *μ*L mixture contained 2 *μ*L of cDNA, 6 pmol each of the forward and reverse primers, and 10 *μ*L of 2 × SuperReal PreMix Plus with SYBR Green I (Tiangen, China). The amplification program was as follows: 95°C for 15 min, 40 cycles at 95°C for 10 s, and 60°C for 32 s. After amplification, a thermal denaturing cycle was added to derive the dissociation curve of the PCR product to verify amplification specificity. Half-quantification of the genes of interest were normalized to Grcc10 and expressed as fold increases over the negative control for each treatment at each time point, as previously described. The primers are as follows: Grcc10 primers: forward 5′- GCGGAGGTGATTCAAGCG -3′ and reverse 5′- TGACCAGGCGGGCAA ACT -3′; influenza A (H1N1) virus M gene Primers: forward 5′-CTGAGAAGCAGATACTGGGC-3′ and reverse 5′-CTGCATTGTCTCCGAAGAAAT-3′.

### 2.11. Measurement of Secretory IgA Levels

In order to collect bronchoalveolar lavage fluids (BALF) and tracheal lavage fluids (TLF), these animals were divided into six groups: normal control group-intubation (NC-I), normal control group-nasal drop (NC-D), normal intubation (NI) group, normal nasal drop (ND) group, model intubation (MI) group, and model nasal drop (MD) group. On the 14^th^ day, tracheal lavage fluids and bronchoalveolar fluids of 5–7 animals in each group were collected and subjected to the enzyme-linked immunosorbent assay (ELISA) to determine secretory IgA [29, 30].

### 2.12. Data Analysis

Data are expressed as mean ± SEM. The statistical analysis of two groups data is calculated by Student's *t*-test. For multiple groups, one-way ANOVA analysis with the LSD test is used to compare means. Analysis involved the use of SPSS 22; statistical significance is considered at ^*∗*^*P* < 0.05 and ^*∗∗*^*P* < 0.01. For survival studies, a log-rank (Mantel–Cox) test involved the use of GraphPad Prism (GraphPad 5.0 Software).

## 3. Results

### 3.1. Establishment of KYDS Mice Model

**Figure 1:**
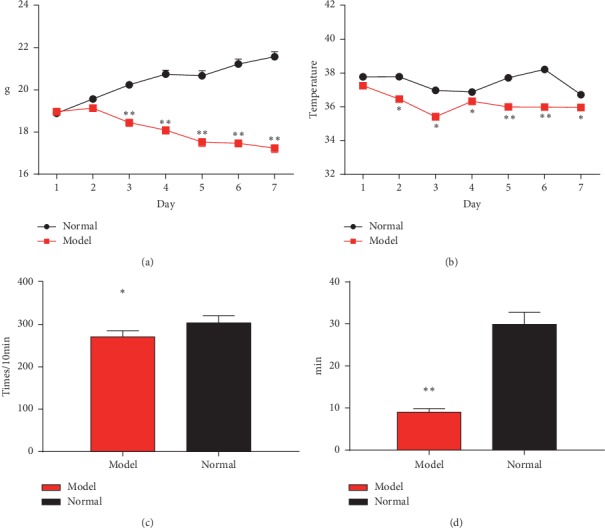
The assessment of the KYDS mice model. Weight of whole body (a) and rectal temperature (b) were measured for establishing the KYDS mice model for seven days. Spontaneous activity (c) and swimming time (d) were calculated in the model group (^*∗*^*P* < 0.05 and ^*∗∗*^*P* < 0.01, compared with the normal control; *n* = 10 in each group).

In order to mimic the high-risk population, we established a KYDS mice model by injecting estradiol benzoate for seven days as previously described; the body weight of the KYDS group was significantly lower than normal control mice from the 3^rd^ day to the 7^th^ day (*P* < 0.01) (Figure 1(a)). The rectal temperature of the KYDS group decreased significantly compared with the normal group for six days (*P* < 0.05) (Figure 1(b)). The spontaneous activity and swimming time of KYDS mice were also obviously lower than the control group (*P* < 0.05; *P* < 0.01) (Figures 1(c) and 1(d)). These results showed that the KYDS mice model was established successfully [21, 22].

### 3.2. Overall Changes in Different Treatment Groups Combined with Influenza A (H1N1) Virus Infection

**Figure 2:**
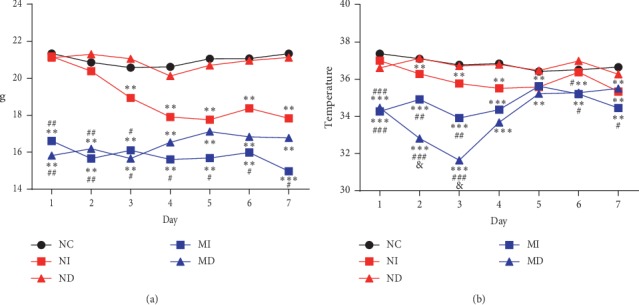
Weight and temperature changes in different treatment groups mice after infection with influenza A virus. Body weight (a) and rectal temperature (b) of the NC group, NI group, ND group, MI group, and MD group were measured for seven days after influenza A virus infection. NI: normal intubation group; ND: normal nasal dropping group; MI: model intubation group; MD: model nasal dropping group; NC: normal control group (^*∗*^*P* < 0.05, ^*∗∗*^*P* < 0.01, and ^*∗∗∗*^*P* < 0.001, compared with the NC and ND groups. ^#^*P* < 0.05, ^##^*P* < 0.01, and ^###^*P* < 0.001, compared with the NI group. ^&^*P* < 0.05, compared with the MI group. *n* = 5–9 in each group).

**Figure 3:**
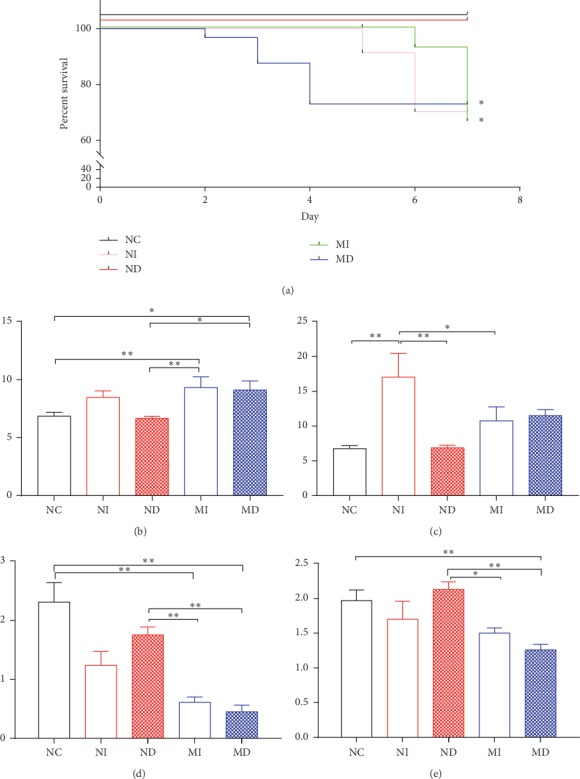
Survival rate and the viscera index of the lung, thymus, and spleen in different treatment group's mice after infection with influenza A virus. Survival proportions of different treatment group's mice after influenza A virus infection were measured for consecutive seven days, and survival analysis was used with the log-rank (Mantel–Cox) test method. Survival rate of the MI group had a significant change compared with the MD or NC group (^*∗*^*P* < 0.05, Figure 3(a)). The viscera index of the lung, thymus, and spleen were compared between varied groups on the 3^rd^ or 7^th^ day (^*∗*^*P* < 0.05 and ^*∗∗*^*P* < 0.01, *n* = 5–9 in each group, Figures 3(b)–3(e)). NI: normal intubation group; ND: normal nasal dropping group; MI: model intubation group; MD: model nasal dropping group; NC: normal control group. (a) Survival propositions. (b) Viscera index of the lung on the 3^rd^ day (c) and on the 7^th^ day. (d) Viscera index of the thymus on the 7^th^ day. (e) Viscera index of the spleen on the 7^th^ day.

Body weight and rectal temperature changes in different treatment group's mice after infection with influenza A virus were measured. NI, MD, and MI groups decreased significantly in weight and temperature compared with the NC and ND groups (*P* < 0.01, Figures 2(a) and 2(b)). The rectal temperature of the MD group reduced significantly from the 2^nd^ to the 3^rd^ day (*P* < 0.01, Figure 2(b)). Survival rate of the MD group decreased obviously from the 2^nd^ to the 4^th^ day. Mortality occurred in the MI and NI group mice after the 5^th^ or 6^th^ day (Figure 3(a)). The viscera index of the lung in the NI and MD groups increased significantly than the NC or ND group on the 3^rd^ day (*P* < 0.01 or *P* < 0.05, Figure 3(b)). The viscera index of the lung in the NI group increased significantly than the NC, ND, and MI group on the 7^th^ day (*P* < 0.01 or *P* < 0.05, Figure 3(c)). The viscera index of the thymus and spleen in the MI and MD groups decreased significantly than the NC or ND group on the 7^th^ day (*P* < 0.01 or *P* < 0.05, Figures 3(d) and 3(e)).

### 3.3. Relative Expression of Influenza A (H1N1) M Genes in Different Treatment Groups

**Figure 4:**
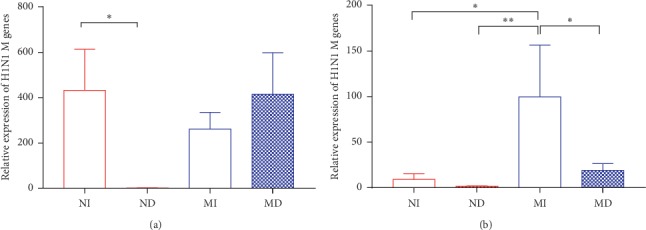
Relative expression of H1N1 M genes in lung tissue in different treatment group's mice after infection with influenza A virus on the 3^rd^ (a) and 7^th^ (b) day (^*∗*^*P* < 0.05 and ^*∗∗*^*P* < 0.01, *n* = 3-4 in each group). NI: normal intubation group; ND: normal nasal dropping group; MI: model intubation group; MD: model nasal dropping group; NC: normal control group.

Relative expression of H1N1 M genes in lung tissue in different treatment groups mice after infection with influenza A virus was measured by RT-qPCR on the 3^rd^ and 7^th^ day. Compared with the ND group, the NI group had a significantly increased expression of influenza A (H1N1) M genes on the 3^rd^ day (*P* < 0.05) (Figure 4(a)). The MD group also had a higher relative M genes level than the MI group, and we could find corresponding experimental results from the survival rate in Figure 3(a), the MD group displayed mortality firstly and seriously. On the 7^th^ day, the MI group had a significant higher expression of influenza A M genes compared with other groups (*P* < 0.05 or *P* < 0.01), and we could also find corresponding results by the survival rate in Figure 3(a), the MI group had the worst survival rate on the 7^th^ day.

### 3.4. Cytokine Expressions in Different Treatment Groups

**Figure 5:**
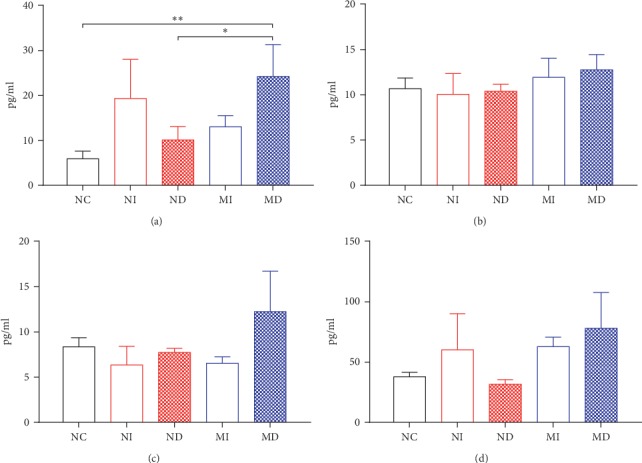
Cytokine expressions in serum in different treatment group's mice after infection with influenza A virus. (a) IL-6, (b) TNF-alpha, (c) IL-12P70, and (d) MCP-1 were measured with the CBA kit (^*∗*^*P* < 0.05 and ^*∗∗*^*P* < 0.01, *n* = 5–9 in each group). NI: normal intubation group; ND: normal nasal dropping group; MI: model intubation group; MD: model nasal dropping group; NC: normal control group.

As seen in Figure 5, compared with the NC or ND group, the MD group increased significantly in IL-6 expression (*P* < 0.01 or *P* < 0.05, Figure 5(a)). Cytokine expressions of IL-12p70, MCP-1, and TNF-alpha did not change significantly between these groups although the MD group had an increasing tendency compared with other groups (Figures 5(b)–5(d)).

### 3.5. SIgA Expressions in Different Treatment Groups

**Figure 6:**
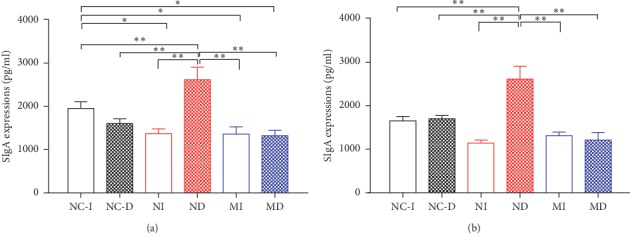
SIgA expression in tracheal lavage fluids (TLF) (a) and bronchoalveolar lavage fluids (BALF) (b) in different treatment group's mice after infection with influenza A virus on the 7^th^ day. NC-I: normal control group-intubation; NC-D: normal control group-nasal drop; NI: normal intubation group; ND: normal nasal dropping group; MI: model intubation group; MD: model nasal dropping group; NC: normal control group (^*∗*^*P* < 0.05 and ^*∗∗*^*P* < 0.01, *n* = 5–7 in each group).

Lavage fluids of mice after infection with influenza A virus were taken on the 7^th^ day. As shown in Figure 6, the ND group increased significantly than all the other groups in SIgA expression in tracheal lavage fluids (*P* < 0.01, Figure 6(a)). Compared with the NC-I group, NI, MI, and MD groups decreased significantly in SIgA expression in tracheal lavage fluids (*P* < 0.05, Figure 6(a)). The ND group also increased significantly than all the other groups in SIgA expression in bronchoalveolar lavage fluids (*P* < 0.01, Figure 6(b)).

### 3.6. Pathological Changes of Lung Tissues

**Figure 7:**
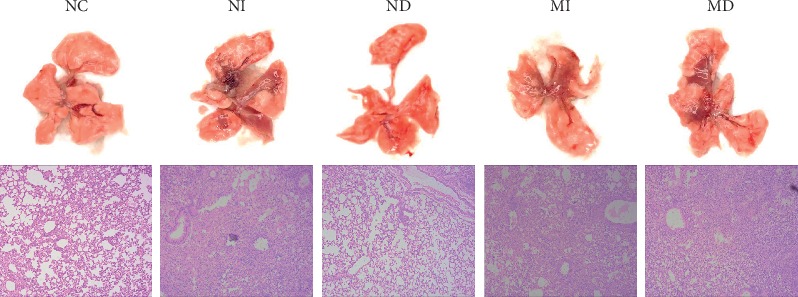
Lung histopathology of different treatment group's mice after infection with influenza A virus. Representative histologic sections of lung tissues from experimental mice were stained by hematoxylin and eosin (magnification, ×100).

Lung tissues and hematoxylin and eosin (H&E) staining of lung sections from BALB/c mice treated with different treatment groups are shown in Figure 7. On the 7^th^ day after influenza A (H1N1) virus infection, no lesions were observed in the lungs of the NC group mice. Lung organs of NI, MI, and MD groups had obviously been characterized by red blood cell extravasation, extensive edema, and inflammation. Pathological changes of the ND group mice lungs were slighter, compared to those of the other infected groups.

## 4. Discussion

Influenza viruses could cause worldwide pandemic and have a serious morbidity and mortality in high-risk groups. Mucosal immunity of the pharynx and larynx is the first defense constitution against viral infection in the host innate immune system. Therefore, further studies in the experiment are needed to decipher the role of mucosal immunity to influenza virus, particularly at the initial site of infection.

As we have mentioned that influenza viral infections mostly have upper respiratory symptoms with sore throat or discomfort symptom, we hypothesized that the mucosal immunity of the pharynx and larynx may play an important role against influenza virus infection in the initial stage in high-risk population, especially. In order to study the mucosal function of the pharynx and larynx, we designed a novel experiment to infect mice with the influenza virus by tracheal injection through a specially manufactured plastic tube, which could ensure influenza virus bypasses the throat part.

In this study, we established a KYDS mouse model with a classic method by intraperitoneally injecting estradiol benzoate as previously described [21, 23] and combined with influenza A (H1N1) virus infection to imitate high-risk patients who were infected with influenza A virus. The KYDS mice and normal mice were separately infected with the influenza A (H1N1) virus through nose dropping and tracheal injection. These data manifested that the KYDS virus group (MI and MD) had a significant decrease in body weights and rectal temperatures compared with normal control mice (Figures 2(a) and 2(b)). An interesting result is that rectal temperatures of the MD group reduced significantly from the 2^nd^ to the 3^rd^ day compared with the MI group (Figure 2(b)). The survival proportions also showed these properties, the MD group had an obviously increased mortality between 2^nd^ and 4^th^ day. These data are obviously different with customary understanding. As our conventional knowledge, the closer the influenza virus infects the lung organ, the more serious the host will be. Otherwise, the NI and ND group changes were expected commonly in body weights and rectal temperatures (Figures 2(a) and 2(b)); the NI group was more serious than the ND group, and the survival proportions also proved it (Figure 3(a)). NI and ND group data are in accordance with customary understanding.

Seasonal influenza A virus infections induce lung injury and extensive inflammation, and inflammatory responses contribute to the morbidity and mortality [31–33]. The viscera index of the lung is an important indication which presents host inflammation in influenza virus infection. On the 7^th^ day after influenza virus infection, the lung index was significantly increased in NI and MD groups compared with NC and ND groups (Figure 3(b)), which manifested that treatment with influenza virus infection in NI and MD groups obviously induced lung inflammation. Lung histopathology also reflected these changes; NI and MD groups had more red blood cell extravasation, extensive edema, and inflammation (Figure 7). It is known that the thymus contains T lymphocytes, while the spleen is the organ containing T and B cells; indices of the thymus and spleen reflect immune function and prognosis of an organism [34]. On the contrary, the thymus and spleen indices reflected immunocompromised function in groups of MI and MD, compared with NC and ND groups (Figures 3(c) and 3(d)). Relative expressions of influenza A (H1N1) M genes displayed that the MD group had a higher level than the MI group on the 3^rd^ day.

IL-6, IL-12p70, MCP-1, and TNF-alpha are cytokines or chemokines which can lead to severe inflammation by influenza A virus infection [35, 36]. Our study also illustrated that cytokine expression of IL-6 in serum illuminated proinflammatory in the MD group compared with the ND or NC group (Figure 5). Cytokine expressions of MCP-1, IL-12p70, and TNF-alpha in serum in the MD group had an increasing trend compared with other groups. These data indicate that the MD group has a more serious inflammation than others.

Secretory IgA (SIgA) is covering mucosal surfaces, which is the major contributor to pathogen-specific immune responses to protect the host from infection [37]. A large number of studies show that SIgA antibodies are present at high levels in the upper respiratory tract, nasal mucus, or nasal swab. Antibody levels determined that induction of SIgA is important for the prevention of infection and protection against influenza [38, 39]. SIgA can protect the murine nose and the upper respiratory tract under normal conditions by preventing viral infection [40]. The mucosal immune responses in the lower respiratory tract are induced in the bronchus-associated lymphoid tissue (BALT), and the ratio of SIgA antibody amount is about 17.4% in BALT to that in the total respiratory tract under conditions of protection against influenza infection. On the contrary, the ratio of SIgA antibody amount in the nasal cavity pharynx is the principal part, the distribution is about 73.6% [39, 41]. In different host conditions, a lot of vaccine research studies have been carried out with elicitation of SIgA antibodies separately. The live virus vaccine is usually approved for the age group of 5–49 years, thus excluding two major high-risk groups, the infants and the elderly. This vaccine can induce SIgA and IgG antibodies, but the live vaccine usually causes rhinitis, sore throat, and fever reactions [42]. However, inactivated vaccines induce mainly serum IgG antibodies rather than mucosal IgA antibodies [43]. These evidences also prove that SIgA antibodies can immediately eliminate a pathogen before the mucosal barrier is passed by the virus; SIgA plays a decisive role in protection against influenza virus infection [44, 45]. But in different host conditions, the distinct protection of SIgA is still unknown, especially in the high-risk population.

In our experiments, we could find some interesting results from lavage fluids (Figure 6). Tracheal lavage fluids (TLF) included throat and a little upper part of the trachea. SIgA antibodies expressions were significantly induced in the ND group compared with the NI group. It reflects that the humoral mucosal immunity of SIgA antibodies is produced mainly in the throat and upper part of the trachea when the host is infected by the influenza A virus. Other evidences also indicate that SIgA plays an effective action in protecting against influenza virus infection of the human upper respiratory mucosa [44–46]. In the present study, our new findings discovered that SIgA antibodies expressions were both decreased significantly in MI and MD groups compared with ND, NC-I, and NC-D groups. Because the model mice (KYDS) are in the lower body resistance condition, productions of SIgA are affected seriously after influenza A virus infection. Subsequent changes of SIgA are similar in bronchoalveolar lavage fluids (BALF). The same changing characteristics of SIgA in the ND group were higher than the NI group; and in MI and MD groups, they were lower than the ND group. Mice treated with nasal dropping of the influenza A (H1N1) virus in the MD group have induced risk of death, suggesting that a decrease in SIgA response may account for unprotecting against severe infection.

We can get a preliminary conclusion the pharynx and larynx could produce enormous SIgA antibodies secretions against influenza virus infection when the body is in normal condition. SIgA can provide immediate immunity response to eliminate the influenza A virus before it passes the mucosal barrier. SIgA antibodies secretions of the lung organ are triggered by the pharynx and larynx organ. When the host is in disease status (for instance in KYDS), SIgA antibodies secretions of the pharynx and larynx organ inhibited for pathogens, and also restrained the SIgA expressions in the lung.

Briefly, the MD group has an earlier and serious damage than the MI group. The intrinsic mechanism is related with SIgA expressions in the pharynx and larynx, which may exerts a decisive role in protection or cross-protection against influenza virus infection in high-risk people condition.

## 5. Conclusions

The present study revealed that SIgA antibody secretions are influenced directly by different conditions of the host in the pharynx and larynx in the upper respiratory tract mucosa. In KYDS virus disease condition, SIgA expressions could be inhibited severely, which leads to serious morbidity and mortality after influenza A virus infection. So the pharynx and larynx exerts a decisive role in protection or cross-protection against influenza virus infection. The SIgA expressions of the pharynx and larynx would be an important target in high-risk populations against influenza A virus infection for vaccine or antiviral drugs research.

## Data Availability

The data used to support the findings of this study are available from the corresponding author upon request.

## Abbreviations

KYDS: Kidney yang deficiency syndrome
NI: Normal intubation group
ND: Normal nasal dropping group
MI: Model intubation group
MD: Model nasal dropping group
NC: Normal control group
H&E: Hematoxylin and eosin
SIgA: Secretory IgA antibodies
TLF: Tracheal lavage fluids
BALF: Bronchoalveolar lavage fluids
TCM: Traditional Chinese medicine
NC-I: Normal control group-intubation
NC-D: Normal control group-nasal drop
IL-6: Interleukin-6
IL-12p70: Interleukin-12p70
MCP-1: Monocyte chemoattractant protein-1
TNF-alpha: Tumor necrosis factor-alpha
RT-qPCR: Reverse transcription quantitative polymerase chain reaction
CBA: Cytometric bead array.
